# Suitable ecological niches of invasive malaria vector under present and projected climatic conditions in South of Iran

**DOI:** 10.1371/journal.pntd.0014054

**Published:** 2026-03-16

**Authors:** Faramarz Bozorg-Omid, Madineh Abbasi, Mohammad Reza Yaghoobi-Ershadi, Ahmad Ali Hanafi-Bojd

**Affiliations:** 1 Navarra Center for International Development (NCID), Institute for Culture and Society, University of Navarra, Pamplona, Spain; 2 Infectious and Tropical Diseases Research centre, Tabriz University of Medical Sciences, Tabriz, Iran; 3 Department of Vector Biology and Control of Diseases, School of Public Health, Tehran University of Medical Sciences, Tehran, Iran; 4 Zoonoses Research Centre, Tehran University of Medical Sciences, Tehran, Iran; National University of Singapore, SINGAPORE

## Abstract

**Background:**

The invasive mosquito species *Anopheles stephensi* plays a critical role in malaria transmission, particularly in urban environments. Its ability to thrive in such settings has raised public health concerns, especially as it expands its geographical range. The resurgence of malaria in Iran underscores the challenges posed by this vector, which is further complicated by factors such as climate change and the movement of populations. Understanding the ecological niches of *An. stephensi* is essential for developing targeted malaria control strategies. This study aims to assess the current and projected distribution of *An. stephensi* in Hormozgan Province, Iran, under varying climatic conditions.

**Method:**

The study was conducted in Hormozgan Province, Iran, characterized by a hot, arid climate. A database of 96 occurrence points for *An*. *stephensi* was compiled through literature searches, which were refined to 72 points to ensure data quality. Environmental and bioclimatic data were sourced from the WorldClim v2.1 database, with a focus on various Shared Socioeconomic Pathways (SSPs). The MaxEnt modeling technique was employed to assess the impact of climate change on the species’ distribution, with model performance evaluated using the two metrics, Area Under the Curve (AUC) and True Skill Statistic (TSS). The analysis aimed to map shifts in suitable habitats under different climate scenarios.

**Results:**

The MaxEnt model predicts a significant decline in environmental suitability for *An. stephensi* under future climate scenarios, particularly in western and central Hormozgan Province. Habitat loss is prevalent, with stable areas primarily located in Bashagard County. Factors such as altitude and precipitation patterns significantly influence species distribution, with altitude showing the highest impact. The model’s performance, indicated by an average AUC of 0.765 and a TSS of 0.519, demonstrates moderate predictive accuracy for identifying suitable habitats.

**Discussion:**

Despite advancements in malaria control, *An*. *stephensi* remains a significant threat in Iran, particularly due to its invasive nature and adaptability to climate change. The study indicates a projected decline in suitable habitats, especially in coastal areas, highlighting the need for adaptive vector control strategies. Bashagard County may serve as a stable refuge, warranting further investigation. The findings emphasize the importance of continuous monitoring and integrating climate projections into public health interventions to effectively combat malaria transmission. Overall, ongoing research is crucial to refining understanding and enhancing malaria control efforts in response to shifting environmental conditions.

## Author summary

The mosquito *An. stephensi* is an important vector of malaria. Unlike many other malaria vectors, it can survive and reproduce in cities, which makes it a growing concern for public health. In recent years, this mosquito has expanded its presence in Iran, raising fears that malaria may spread again, especially as climate change creates new challenges for disease control. In this study, we explored how current and future climate conditions might affect where *An. stephensi* can live in Hormozgan Province, southern Iran. We used climate data and records of where the mosquito has been found to create maps showing areas that are most suitable for its survival. Our results suggest that many areas, especially in central and western Hormozgan, may become less suitable for the mosquito in the future. However, some regions, particularly Bashagard County, are likely to remain favorable habitats. These findings can guide health authorities in designing more effective and adaptive strategies to reduce malaria transmission.

## Introduction

In 2023, the World Health Organization (WHO) reported 263 million new cases of malaria globally [1]. Iran effectively executed malaria control measures, resulting in 2021 having no indigenous malaria cases for 4 consecutive years [2]. Nevertheless, a resurgence of the disease was observed in 2022, with confirmed malaria cases rising sharply to around 1,432 locally acquired infections that year (total cases: 5,677). Furthermore, in 2023, the caseload doubled compared to the previous year (total cases: 9,868) [3,4]. Public health officials attribute the outbreaks to several factors, including the presence of foreign nationals, an increase in malaria cases in neighboring Pakistan, poor detection of new cases, heavy summer rains, a focus on the COVID-19 pandemic, and the emergence of invasive *Aedes* species. This emergence has diverted the healthcare system’s attention and resources away from malaria control efforts, leading to increased vulnerability and resulting in the local transmission of the newly emerged dengue fever in Iran [5]. The regions most impacted in Iran are the south and southeast [3,6].

*Anopheles stephensi*, a mosquito species originally from the Indian subcontinent and the Persian Gulf, is notable for its role in transmitting malaria parasites, specifically *Plasmodium falciparum* and *P. vivax*. Unlike other malaria vectors, it thrives in urban environments and has significantly expanded its range westward since its first detection in Africa in Djibouti in 2012. This species has since been reported in several countries across the Horn of Africa, including Ethiopia, Sudan, Somalia, and Eritrea, as well as in Yemen, Kenya, Ghana, and Nigeria, highlighting its growing geographical distribution and public health implications [7–13]*.* Recent habitat suitability studies provide further insights into this expansion: Sinka et al. (2020) used spatial models to predict potential urban areas across Africa where *An. stephensi* could establish, estimating that over 126 million people may be at risk if the spread continues unchecked [14]. Similarly, Samake et al. (2025) analyzed the distribution and genetic diversity of *An. stephensi* in Kenya, revealing multiple introductions, co-occurrence with native *Anopheles* species, and environmental factors such as low precipitation and minimal seasonal temperature variation influencing its spread, underscoring the need for ongoing surveillance and predictive modeling [15].

The influence of global climate change on natural ecosystems has been extensively documented, highlighting its profound effects on human welfare and biodiversity [16–18]. The latest valuation report from the United Nations Intergovernmental Panel on Climate Change (IPCC AR5) highlights a significant increase in global land and ocean temperatures, with a rise of 0.89 °C (0.69-1.08 °C) from 1901 to 2012 [19]. This temperature increase is anticipated to have profound implications, including potential changes in suitable ranges for species and the exacerbation of the negative impacts of invasive species in various regions [20,21]. Climate change also affects the El Niño cycle, impacting the transmission of diseases like malaria, dengue, and Rift Valley fever [22]. Moreover, the complex connection between the geographical distribution of malaria, local vectors, and climatic and geographic factors is becoming increasingly clear, directly impacting the local epidemiology of malaria [23,24]. This emphasizes the need to integrate climate change considerations into malaria control strategies [23,25,26].

Efforts to control malaria through surveillance programs, including early diagnosis, treatment, and prevention measures (such as insecticide-treated nets), have shown significant progress in reducing malaria transmission rates [27,28]. The Global Malaria Action Plan aims to eliminate malaria in several countries. However, the impacts of climate change may pose considerable challenges to these efforts, potentially increasing the epidemic potential of malaria in susceptible regions [29,30]. Therefore, maintaining strong surveillance and preparedness is crucial, especially in developing countries with limited resources. As global temperatures continue to rise, the range and behavior of malaria vectors, such as *An. stephensi* are likely to be significantly affected, potentially leading to shifts in their distribution and posing new challenges for malaria control and elimination efforts [31]. Additionally, a study in the Ecuadorian Amazon reveals that changes in land use and land cover, alongside climate factors, significantly influence malaria risk. This indicates that both land use and climate factors must be considered to fully understand malaria dynamics [32].

The use of bioclimatic data and Geographic Information System (GIS) analysis has become fundamental in predicting the potential habitats of insect vectors across time. Climate change scenarios play a crucial role in understanding how future environmental conditions may alter vector distributions [33,34]. Unlike the earlier Representative Concentration Pathways (RCPs), which focused solely on greenhouse gas (GHG) emission trajectories, Shared Socioeconomic Pathways (SSPs) integrate both climate and societal factors, providing a more comprehensive framework for analyzing future environmental and health-related challenges [35,36]. In the context of vector-borne diseases, including malaria, SSPs are crucial because they allow researchers to model not only how climate change may affect the distribution of disease vectors, but also how socioeconomic conditions may influence transmission, control measures, and adaptation strategies [37].

The SSP framework, when combined with Species Distribution Models (SDMs), provides a valuable tool for predicting the future distribution of malaria vectors under different climate and socioeconomic conditions. SDMs, also known as Ecological Niche Models (ENMs), are widely used to predict the geographic range of species under different environmental conditions [38,39]. These models utilize species occurrence records and environmental variables to identify areas with a high probability of species presence [40]. SDMs have proven particularly useful in assessing the potential spread of invasive species and understanding how climate change may alter habitat suitability [41]. Over the past decade, more than 35 modeling approaches have been developed for generating SDMs, each with its advantages and limitations [39]. Some of the most commonly used models include Maximum Entropy (MaxEnt), Generalized Linear Model (GLM), Random Forest [42], and Generalized Boosting Model (GBM). Among these, MaxEnt has been widely recognized for its effectiveness in modeling species distributions using presence-only data. Comparative studies suggest that MaxEnt often performs as well as, or better than, other methods in predicting suitable habitats for species, particularly in cases where occurrence data are limited [38,39,43,44].

High-resolution, region-specific modeling enhances the reliability of predictions, allowing for better-targeted vector control strategies and public health interventions [45]. By incorporating detailed local environmental and climatic data, these models provide valuable insights into future vector distribution trends, aiding in proactive disease management and mitigation efforts [45]. This approach facilitates evidence-based decision-making for malaria prevention and control strategies, ensuring that interventions are targeted toward the most vulnerable regions [45]. Quantitative modeling techniques rooted in ecological niche theory are primarily utilized to understand the relationship between species and their environment [46]. Ecological niche modeling forecasts a species’ range by leveraging its occurrence data and specific environmental parameters to define its niche within an ecosystem, which is then projected onto geographical landscapes [47]. When focused on organisms, it identifies suitable habitats for each species in areas lacking mosquito population data, enabling evidence-based assessments of malaria transmission risk in regions not currently covered by interventions [48,49]. Forecasting the presence of a vector in particular areas poses a difficulty for numerous disease control initiatives aiming to enhance the planning and execution of control strategies and adaptation measures [50]. The maximum entropy model (MaxEnt v3.4.1) [51] is a species distribution prediction model grounded in the maximum entropy principle that exhibits notable predictive performance compared to various other models for predicting species distribution, particularly in scenarios where species distribution models have been suggested to be accurate with small samples [52]. It often outperforms similar prediction models in such cases [53,54].

The objective of this research was to gather all records of *An. stephensi* occurrence in Hormozgan Province, South of Iran, over the past five decades. Additionally, the study aimed to forecast the present and future distribution as well as the environmental suitability of these species for the years 2030s, 2050s, and 2070s. Understanding the spatial distribution pattern of these vectors is essential for evaluating disease transmission risk in various regions and for designing effective vector control programs.

## Materials and methods

### Ethics statement

The protocol for this project has been approved by the ethics committee at the Research Deputy of Tehran University of Medical Sciences, Tehran, Iran, with Ethics Code: IR.TUMS.SPH.REC.1397.297.

### Study area

**Fig 1:**
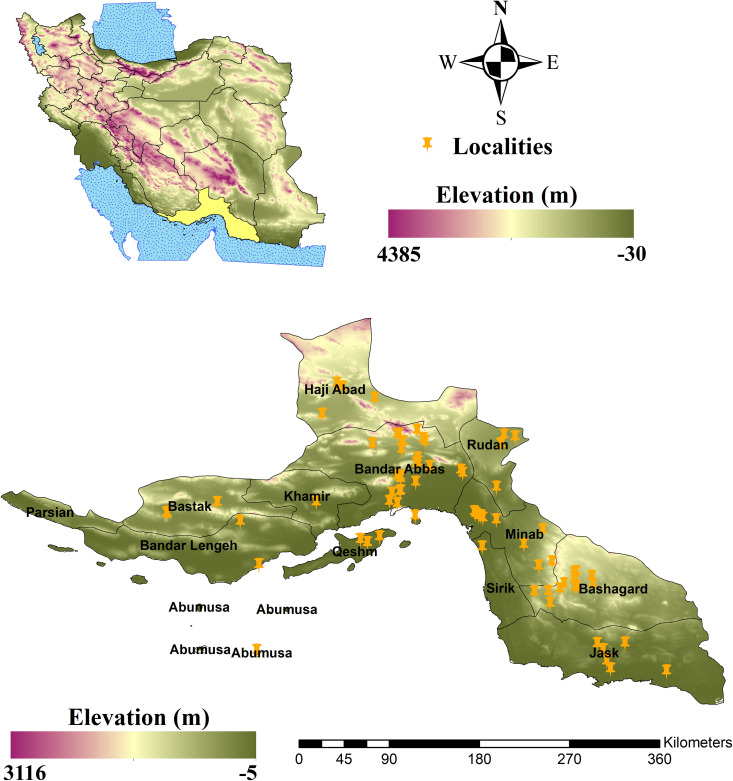
Study area and spatial distribution of *Anopheles stephensi* occurrence points in Hormozgan Province, Iran. Map created using ArcGIS Desktop (version 10.5; Environmental Systems Research Institute, Redlands, CA, USA) (www.esri.com).

Hormozgan Province, situated in the southern region of Iran between 25°23’ and 28°57’ N, and 52°41’ and 59°15’ E, covers an area of 70,000 km^2^. It is bordered by the coasts of the Oman Sea and the Persian Gulf, spanning approximately 900 Km in the south (Fig 1). The climate of the province falls within the subtropical category, characterized by warm air as the most prominent feature. Known as one of the hot and arid regions of Iran, it is heavily influenced by semi-desert and desert climates. Summers along the coastal strip are extremely hot and humid, with temperatures occasionally exceeding 52℃. The average annual temperature stands at 27℃. The province experiences a protracted hot season lasting nine months, starting from early March and reaching its peak in July and August. This is followed by a brief three-month cool season, beginning in early December and influenced by cool western air masses. Topographically, the elevation of Hormozgan Province varies considerably, ranging from 5 m below sea level in coastal areas to 3,116 m in the northern highlands. Overall, the climate of Hormozgan Province closely resembles that of desert areas, with extremely low levels of atmospheric precipitation, resulting in a notable lack of vegetation [55].

### Occurrence data

To compile a presence location database of *Anopheles stephensi*, the terms “*An. stephensi*” and “malaria” were used in a literature search of various online scientific sources (Google Scholar, PubMed, and Web of Science, SID, Irandoc, and Magiran) from 1970 to 2022 (S1 Table). Geographic coordinates were extracted. Following this web search, a total of 96 presence points of *An. stephensi* species were collected. The dataset included both larval and adult detections. To ensure data quality, duplicate records, defined as entries with the same geographic coordinates reported, were removed [56,57]. Additionally, to minimize spatial autocorrelation and prevent pseudo-replication [56], the data were processed using the spatial rarefaction tool in SDMs Toolbox v2.5 (ArcGIS 10.5) and then imported into ArcMap 10.5 for visualization. Finally, the number of points of presence was reduced to 72 (Fig 1) (S1 File).

### Environmental and bioclimatic data

Both topographic (altitude) and bioclimatic data (current (1970–2000) and future (2020–2040, 2041–2060, and 2061–2080) were obtained from the WorldClim v2.1 database (www.worldclim.org) with a spatial resolution of approximately 1 km² [58]. We selected three Shared Socioeconomic Pathways (SSP 1–2.6, SSP2-4.5, and SSP 5–8.5) from the sixth version of the Model for Interdisciplinary Research on Climate (MIROC6), representing scenarios with low, moderate, and high greenhouse gas emission concentrations, respectively. These scenarios were chosen to capture a range of plausible future climate conditions, allowing assessment of how different levels of climate change may influence the distribution and habitat suitability of *An. stephensi* in Hormozgan Province. All bioclimatic and topographic layers in raster format were imported into ArcGIS 10.5, and the data were clipped using the Hormozgan Province boundary shapefile for regional analysis. To ensure the accuracy and reliability of the analysis, we accounted for multicollinearity among environmental variables. Highly correlated climate variables can introduce biases in statistical models, leading to misleading results. Therefore, collinearity among climatic variables was assessed using the Variance Inflation Factor (VIF) method in the *usdm* package in R software [59,60]. Variables with a VIF greater than 10 were removed to minimize redundancy and enhance model performance [59]. A final set of 10 uncorrelated variables was selected from an initial pool of 20 layers (19 bioclimatic variables and one altitude layer) for modeling (Table 1).

**Table 1:** List of environmental variables used in *Anopheles stephensi* distribution modeling, after multicollinearity testing.

Abbreviations	Variables
Bio 3	Isothermality: (Mean Diurnal Range/Temperature Annual Range) × 100
Bio 4	Temperature seasonality (standard deviation × 100)
Bio 8	Mean Temperature of Wettest Quarter
Bio 9	Mean Temperature of Driest Quarter
Bio 10	Mean Temperature of Warmest Quarter
Bio 14	Precipitation of Driest Month
Bio 15	Precipitation seasonality (coefficient of variation)
Bio 18	Precipitation of Warmest Quarter
Bio 19	Precipitation of Coldest Quarter
Alt	Altitude (m)

### Species distribution modeling

The MaxEnt modeling technique was employed to assess the effects of climate change on the distribution shifts of *An. stephensi* in Hormozgan Province [61]. MaxEnt v3.4.3 software was utilized for the analyses. Customized settings were applied for the modeling process, including the selection of 10,000 background points. To evaluate model performance, 80% of the occurrence data were designated for model training (calibration), while the remaining 20% were reserved for model testing (evaluation) with 10 repetitions to ensure robustness of the results. To assess the relative importance of environmental variables in the MaxEnt model, we applied the Jackknife test. This method evaluates each variable individually and in combination with others to determine its contribution to model performance [62].

To illustrate changes in suitable habitat, we qualitatively and quantitatively summarized the distributional shifts in species ranges based on SSP scenarios and time periods. Specifically, “stable”, “gain”, and “loss” areas in species distribution were mapped under future climate change scenarios. The analysis was conducted using R-4.4.2v software, where the continuous habitat suitability outputs were converted into binary presence–absence maps using the Maximum training sensitivity plus specificity (MaxSSS) threshold [63]. This thresholding rule is widely recommended because it provides an optimal trade-off between omission errors (false negatives) and commission errors (false positives), thus improving the ecological realism of the predictions [64]. The gain/loss/stable areas were derived by comparing binary maps with the *terra* package [65]. This approach allowed for a clear representation of the changes in suitable habitats under different climate scenarios. In other words, it shows how habitat suitability is expected to shift under different climate scenarios, whether the environmental suitability has increased, decreased, or remained stable compared to the current conditions. Maps were visualized using the *ggplot2* package [66].

### Model evaluation

Various metrics have been proposed to evaluate the performance of habitat suitability models [44]. However, there is no consensus on which metrics are most appropriate for assessing model performance. Therefore, we employed two widely used metrics to evaluate the generated habitat suitability model: TSS [67,68] and Area Under the Receiver Operating Characteristic Curve (AUC) [69]. AUC values were obtained directly from MaxEnt outputs, while TSS values were calculated using the *presenceAbsence* package in R-4.4.2v software [70]. According to previous studies, TSS ranges from −1 to +1, where +1 represents perfect model performance and a value of 0 indicates predictions no better than random. AUC values range from 0 to 1, with 0.5 indicating random performance and values approaching 1.0 reflecting increasingly better model performance [71,72]. Maps were visualized using the *ggplot2* package [66].

## Result

### Environmental suitability and area gain and loss patterns

**Fig 2:**
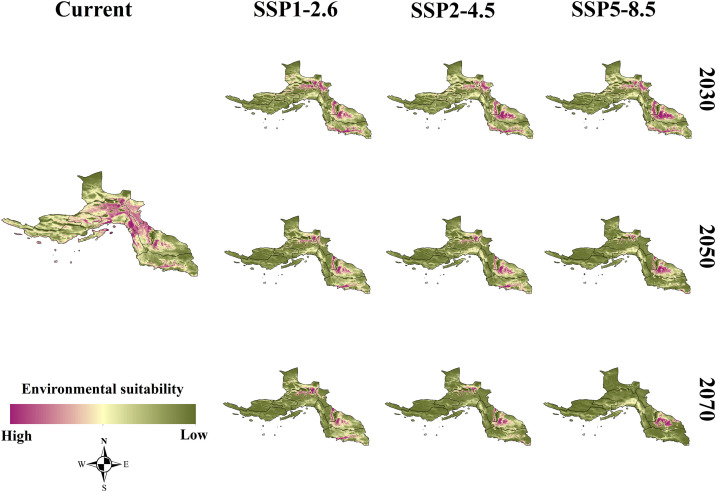
Environmental suitability of the *Anopheles stephensi* species under current climatic conditions in Hormozgan province, Iran. Map created using ArcGIS Desktop (version 10.5; Environmental Systems Research Institute, Redlands, CA, USA) (www.esri.com).

The MaxEnt model projected that environmental suitability for *An. stephensi* declines significantly under future climate change scenarios. This decline becomes progressively more severe over time. The findings suggest that by the end of the century, a large portion of currently suitable areas will no longer provide optimal environmental conditions for the species. The most notable changes in environmental suitability occur in the western and central parts of the province, where Bandar Abbas, Bandar Khamir, and the islands of Qeshm, Kish, and Abu Musa exhibit a sharp decline in suitability. These areas are expected to become increasingly unsuitable due to changing climatic conditions, as reflected in the progressive reduction of suitable habitat patches over time. Conversely, suitable habitats in Bashagard County would be stable areas. Notably, under SSP5-8.5-2070s, Bashagard is the only county that will remain suitable (Fig 2).

**Fig 3:**
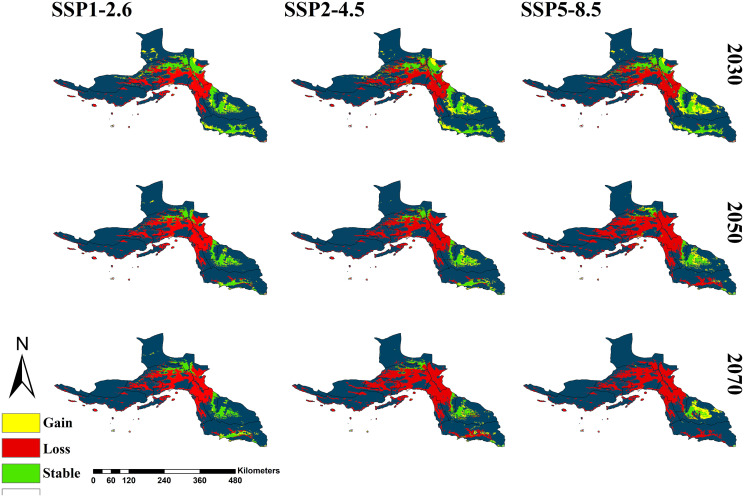
Gain, loss, and stable maps for *Anopheles stephensi* under three climate change scenarios in the 2030s, 2050s, and 2070s in Hormozgan Province, Iran. The Map was generated using R-4.4.2v, with raster processing performed using the *terra* package and visualization using the *ggplot2* package.

The gain and loss maps provide further insights into the spatial redistribution of suitable habitats. The overall trend points to a declining habitat suitability in most of the province, with only a few areas showing potential for habitat gain. Red-colored areas indicate habitat loss, meaning that these regions are currently suitable but are expected to become unsuitable in the future. This is particularly evident in the western and central regions. On the other hand, the gain areas are observed primarily in the eastern and southern parts of the province, particularly in Bashagard. These areas exhibit an expansion of suitable habitats over time, particularly under the SSP5-8.5 scenario, which projects the highest levels of habitat gain. Conversely, the SSP1-2.6 scenario consistently predicts the least habitat gain across different time periods, suggesting that lower-emission scenarios may result in fewer spatial shifts in habitat suitability (Fig 3).

**Fig 4:**
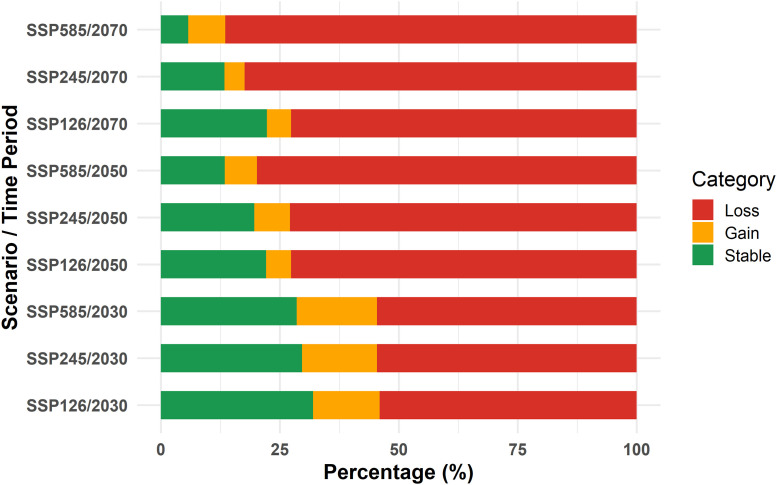
Percentage scaling of gain, loss, and stable areas based on different scenarios in the 2030s, 2050s, and 2070s. The Y-axis shows the climate scenarios (SSP1-2.6, SSP2-4.5, SSP5-8.5) in different periods, and the X-axis shows the percentage of habitat categorized as gain, loss, or stable. Colors indicate habitat change: green = stable, yellow = gain, and red = loss. The Map was generated using R-4.4.2v, with visualization using the *ggplot2* package.

The bar chart illustrates the proportions of Stable, Gain, and Loss across different SSP scenarios. The dominant category in all SSPs is Loss, consistently occupying the largest portion, ranging between approximately 55% and 85%. The highest percentage of loss is seen in the 2070s and 2050s, especially in the SSP5-8.5 and SSP2-4.5 scenarios, each having nearly 75–85% of the total. The Stable category varies significantly, with the 2030 period, especially SSP1-2.6, showing the highest stability at around 30%. The Gain category remains relatively small in all cases, with percentages generally ranging between 5% and 15%. The highest gain percentage appears in the 2030s and the SSP5-8.5 scenario, where it reaches around 15%. On the other hand, the lowest stability is observed in the 2070s and the SSP5-8.5 scenario, with only about 5% remaining stable. Overall, the chart emphasizes that most scenarios experience overwhelming loss, with only minor portions remaining stable or gaining (Fig 4).

**Fig 5:**
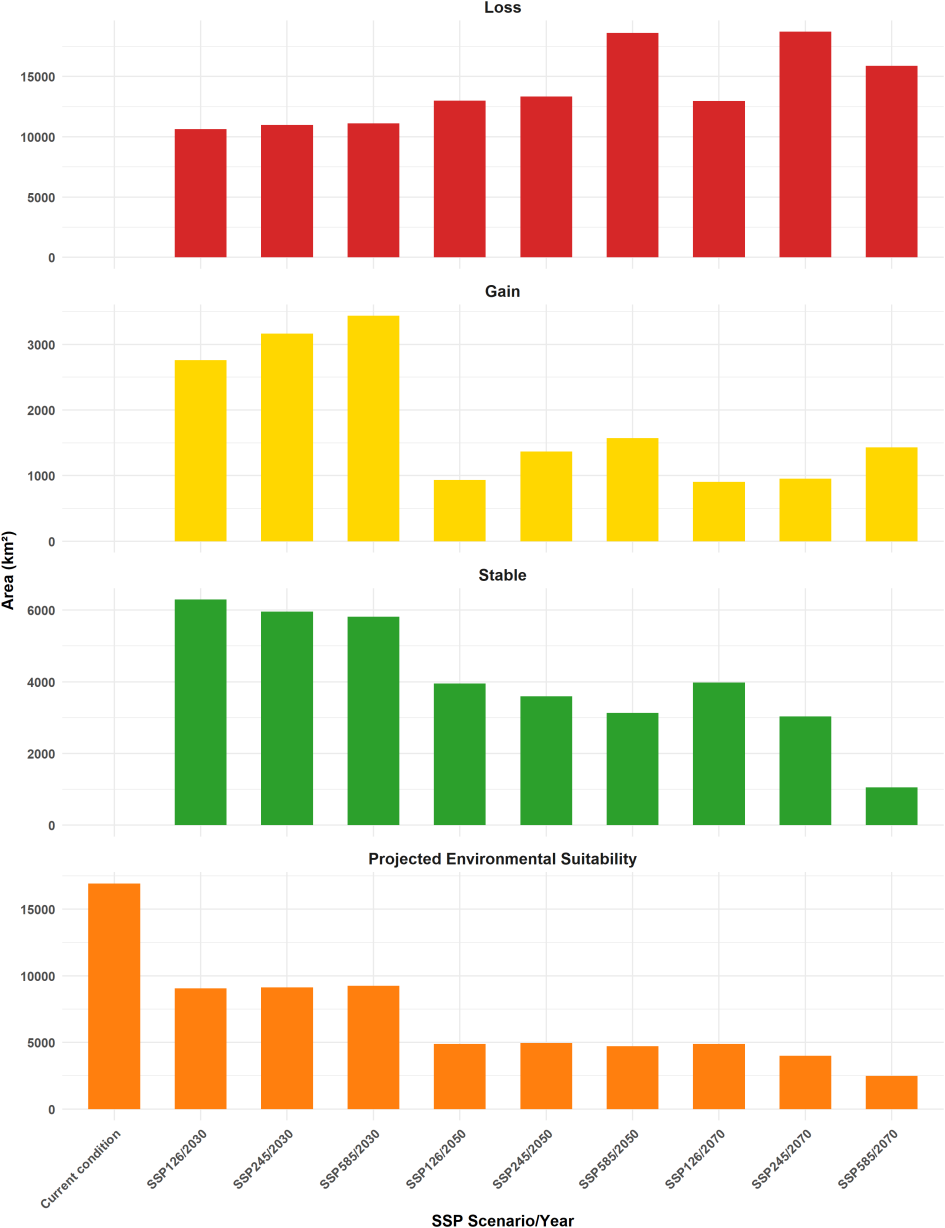
Area of projected environmental suitability, gain, loss, and stable habitat based on various climate scenarios and time periods, shown in square kilometers. Projected environmental suitability: suitable area (km²) under current conditions and three climate scenarios (SSP1-2.6, SSP2-4.5, and SSP5-8.5) for 2030, 2050, and 2070; Gain, Loss, and Stable: areas of habitat expected to increase, decrease, and areas expected to remain stable, respectively, under the same scenarios, in comparison with environmental suitability. The Map was generated using R-4.4.2v, with visualization using the *ggplot2* package.

An analysis of changing suitable areas, measured in square kilometers, across various SSP scenarios reveals significant trends in environmental suitability, stability, gain, and loss areas over time. The projected environmental suitability decreases, starting at approximately 9,200 km^2^ in the 2030s and dropping to 2,490 km^2^ in the 2070s, SSP5-8.5 scenario. Stability consistently declines from 6,300 km^2^ in the 2030s, SSP1-2.6 scenario, to 1,052 km^2^ from the 2070 period (SSP5-8.5 scenario). Although gain areas initially peak at approximately 3,500 km^2^ in SSP5-8.5 from the 2030s, they diminish significantly in subsequent scenarios, reaching a low of 900 km^2^ in the 2070s, SSP1-2.6 scenario. Conversely, loss areas increase over time, beginning at approximately 10,635 km^2^ in the SSP1-2.6 scenario from the 2030 period and rising to 18,704 km^2^ in the 2070s, SSP2-4.5 scenario. This indicates a pattern of decreasing suitability, stability, and gain, with an increase in environmental loss, especially in more extreme scenarios (Fig 5).

### Analysis of variable contributions

**Fig 6:**
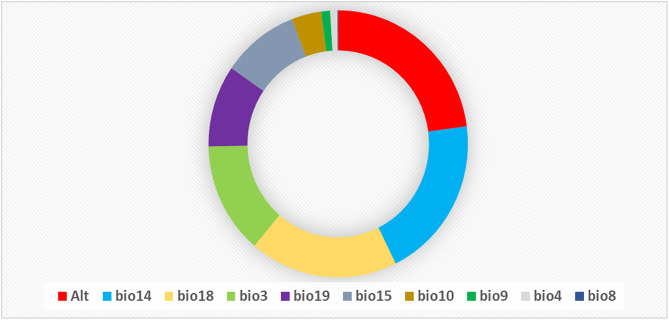
Relative importance of environmental variables to the MaxEnt model. Bio3 = Isothermality ((Mean Diurnal Range/ Temperature Annual Range) ×100), Bio4 = Temperature seasonality (SD × 100), Bio8 = Mean Temperature of Wettest Quarter, Bio9 = Mean Temperature of Driest Quarter, Bio10 = Mean Temperature of Warmest Quarter, Bio14 = Precipitation of Driest Month, Bio15 = Precipitation seasonality (CV), Bio18 = Precipitation of Warmest Quarter, Bio19 = Precipitation of Coldest Quarter, Alt = Altitude **(m)**.

**Fig 7:**
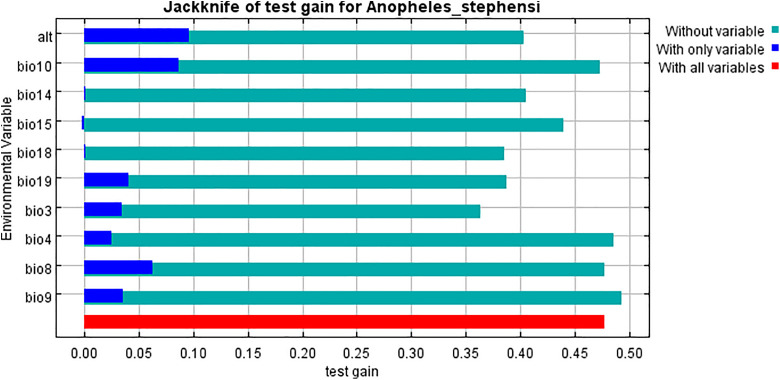
Jackknife analysis of environmental variable importance in the MaxEnt model. The Y-axis shows environmental variables, and the X-axis represents model gain during testing. Colors indicate the contribution of each variable: green = variable excluded from the model, blue = model with only that variable, red = model with all variables included. Variables with taller blue bars demonstrate strong predictive power when used alone, while shorter green bars indicate that excluding the variable substantially reduces the model’s predictive accuracy, highlighting its critical importance.

Based on the permutation importance values in Fig 6, ALT (altitude) has the highest impact on the model with 22.9%, followed closely by bio14 (precipitation of the driest month) at 19.8% and bio18 (precipitation of the warmest quarter) at 18.5%. bio3 (isothermality) also plays a significant role with 13.5%, while bio19 (precipitation of the coldest quarter) and bio15 (precipitation seasonality) have moderate importance at 9.9% and 9.6%, respectively. Other variables, such as bio10 (mean temperature of the warmest quarter) and bio9 (mean temperature of the driest quarter), have lower permutation importance at 3.8% and 1.1%, while bio8 (mean temperature of the wettest quarter) has the least impact with only 0.1% (Fig 6). These results indicate that certain environment-related variables strongly influence the model’s predictions. Based on the jackknife results using test gain, ALT (altitude) stands out as the single most informative variable when used alone, indicating it provides substantial predictive power by itself. Meanwhile, bio3 (isothermality) leads to the greatest decrease in gain when omitted, suggesting it offers unique information not captured by the other variables (Fig 7).

### Response curves

**Fig 8:**
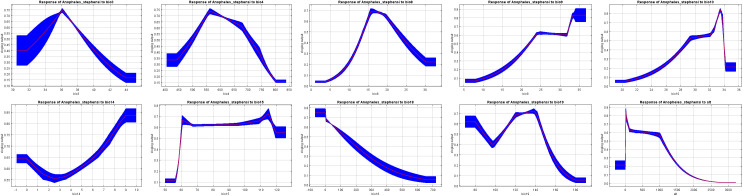
Response curves of the environmental variables that contributed to the MaxEnt model. The curves (blue) show the mean response of the 10 replicate MaxEnt runs [8] and the mean + /- one standard deviation. The x-axis shows environmental variables (see Table 1), and the y-axis denotes the predicted probability of species presence on the cloglog scale, where cloglog transforms the model output into an occurrence probability between 0 and 1.

Curves in Fig 8 show how each environmental variable is associated with the predicted probability of *An. stephensi* presence. The response curve analysis indicates that higher temperatures and precipitation levels are predicted to be associated with higher probabilities of the presence of *An. stephensi*. Among these factors, altitude shows an inverse relationship with the predicted probability of presence, with the highest predicted probability recorded at 200 meters. Isothermality (Bio3) varies between 32% and 44%, with the predicted probability peaking at 36% before declining. The mean temperature of the warmest quarter (Bio10) is predicted to influence the probability of presence, fluctuating between 20°C and 34°C, with the highest predicted probability at 33°C. Similarly, the mean temperature of the wettest quarter (Bio8) follows the same positive association, ranging from 5°C to 30°C, with a peak predicted probability at 17°C. Precipitation of the driest month (Bio14) falls within -1 mm to 9 mm, where the predicted probability of presence increases beyond 3 mm, peaking at 9 mm. Precipitation of the coldest quarter (Bio19) ranges between 80 mm and 180 mm, with the highest predicted probability observed at 140 mm. Conversely, precipitation of the warmest quarter (Bio18) is predicted to be associated with lower probabilities of presence, varying from 0 mm to 700 mm. Precipitation seasonality (Bio15) fluctuates between 55 and 120, with predicted probabilities showing two peaks at 60 and 115. Temperature seasonality (Bio4) ranges from 500 to 800, with the highest predicted probability at 550 (Fig 8).

### Model performance

**Fig 9:**
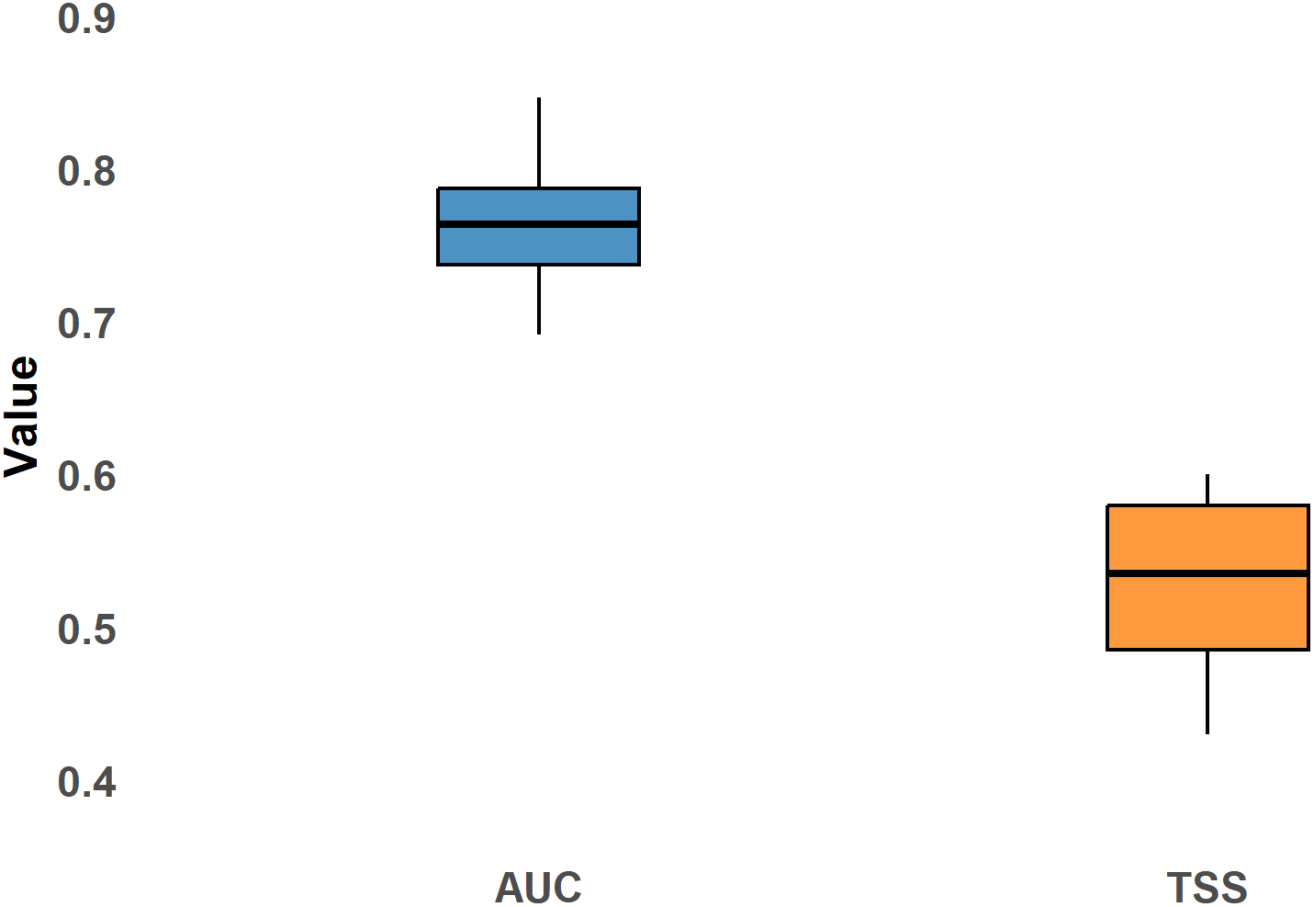
TSS and AUC metrics for model performance. The Map was generated using R-4.4.2v, with visualization using the *ggplot2* package.

The model’s predictive performance was evaluated using the TSS and the AUC. Across ten replicate runs, the average TSS was 0.519, while the average AUC was 0.765, indicating consistent model performance (Fig 9).

## Discussion

Despite significant progress in malaria control measures over the past decades, malaria remains a persistent problem in the southern provinces of Iran [4]. Monitoring vectors is a crucial aspect of planning, executing, and assessing vector control measures. Gaining a comprehensive understanding of the distribution of malaria vector populations across a country is an invaluable asset in combating malaria transmission [73]. With global temperatures rising, understanding and mitigating the impact of climate change on malaria vectors is crucial to ensuring effective public health measures and preserving natural ecosystems. Among malaria vectors, *An. stephensi* stands out due to its invasive nature and its ability to thrive in rapidly changing environments [10,11]. Its growing presence in new regions underscores the urgent need for continuous entomological surveillance and proactive vector control strategies. Given the dynamic nature of malaria vectors and the influence of climate change on their distribution, predictive modeling can offer valuable insights for designing effective control strategies. In this study, we employed species distribution modeling using MaxEnt to assess the current and future distribution of *An. stephensi* in Hormozgan Province, Iran, providing valuable insights into how climate change may reshape its habitat suitability.

Our projection indicates that habitat loss is the dominant trend for *An. stephensi* across all SSP scenarios, with a particularly sharp decline observed under the SSP5-8.5 scenario in the 2070s. Conversely, in colder regions, climate change is expected to increase the suitability of habitats for this species. Studies have predicted that a warming temperature is enabling *An. stephensi* to thrive in once inhospitable areas, thereby expanding their geographic range [74,75]. With climate change and rising temperatures, the thermal conditions suitable for this species are likely to shrink further, reducing habitat suitability in Hormozgan. In contrast, in cooler regions of the world, warming may expand the potential range of this vector, highlighting the context-dependent effects of climate change on *An. stephensi* distribution [74,75]. Therefore, it is crucial to consider regional variations when assessing the future risks associated with climate change. The projected reduction of suitable habitats in Bandar Abbas, Bandar Khamir, and the islands of Qeshm, Kish, and Abu Musa suggests that coastal and low-altitude areas (Fig 3) may become increasingly unsuitable. Conversely, Bashagard County, despite being lowland, appears to retain more stable environmental conditions and may serve as a potential refugium for *An. stephensi*. This resilience suggests that specific environmental conditions in Bashagard may buffer against climate change effects, warranting further investigation into its microclimatic and ecological characteristics. The species exhibits notable ecological plasticity, being able to exploit a variety of domestic and peri-domestic habitats, including storage tanks, overhead tanks, and discarded containers [76,77]. However, the reliance on human-made water containers also implies that anthropogenic factors, such as urbanization, water management practices, and waste disposal, will strongly influence local population dynamics. Consequently, even in regions where climatic conditions become less favorable, the species may maintain viable populations if suitable artificial habitats are available. Nonetheless, the species remains sensitive to environmental conditions, particularly temperature, which can influence its survival and reproduction. Previous studies in Iran have shown that *An. stephensi* is sensitive to temperature, surviving within a range of approximately 10.3 °C to 38 °C and requiring around 187 cumulative degree-days for development [78]. These physiological thresholds may help explain why some areas remain suitable for the species despite broader climatic changes. While climate change may create some new suitable habitats, especially under high-emission scenarios like SSP5-8.5, the MaxEnt model estimated the loss area as more than the gain area for *An. stephensi* in Hormozgan province. So that the total area of suitable habitats drops significantly over time from 6,300 km² in the 2030s to just 1,052 km² in the 2070s under SSP5-8.5. Furthermore, as the species may shift to higher latitudes [79] or neighboring provinces, further research is essential, especially in southern provinces, to assess the potential impacts of new SSP scenarios on habitat availability and vector control strategies.

Moreover, projections show that suitable habitats for most malaria vector species in Iran are expected to decline in the future [80–82]. However, global trends present a more complex picture. While studies predict an expansion of malaria vectors in China and Central America in the coming decades [83,84], the availability of suitable habitats for these vectors is expected to decline in regions such as Africa and South America [85]. Previous studies have generally suggested that habitat suitability for *An. stephensi* remains relatively stable under climate change in Iran [80,81,86]. In contrast, our findings indicate a significant decline in suitability over time. This difference highlights the importance of conducting localized studies at smaller spatial scales because national-scale assessments might overlook region-specific variations and more detailed ecological characteristics. Additionally, another potential reason for these contrasting results is the use of different climate scenarios. All of the previous studies in Iran have relied on RCP scenarios [80,81,86], whereas our study employs SSP scenarios. Unlike RCPs, SSPs incorporate both climate projections and socio-economic factors, which may provide a more comprehensive assessment of future environmental suitability. In other words, SSP scenarios have emerged as advanced climate modeling frameworks that account not only for greenhouse gas concentration levels but also for the broader socio-economic drivers of climate change, including population dynamics, economic development, land use patterns, and energy transitions [35]. Moreover, the referenced studies did not account for potential correlations between climatic variables and incorporated all 19 layers in their analysis. Additionally, discrepancies in findings may stem from differences in the selected global climate models (GCMs), as each model produces distinct regional climate change projections. In our study, we utilized the SSP-MIROC6 model, whereas previous studies were based on the RCP-BCC-CEM2-MR model, which may have contributed to the observed variations in habitat suitability predictions. These methodological differences may partly explain the contrasting results, underscoring the need for continued research at multiple spatial scales to ensure accurate assessments of vector distribution dynamics [87].

As highlighted by Ryan et al. (2023), the anticipated spread of this vector necessitates proactive monitoring and targeted strategies to mitigate transmission risks in areas projected to become suitable under future climate scenarios. This underscores the importance of ongoing entomological surveillance, as some studies suggest that climate change may not uniformly affect vector distribution, indicating a complex relationship of ecological factors that permit further investigation [86–88]. Understanding the shift in malaria patterns due to changes in habitat suitability requires a comprehensive approach that accounts for multiple epidemiological factors. While habitat change may influence vector distribution, it is crucial to recognize that malaria transmission in this province is not solely driven by a single vector species. Previous studies have shown that *Anopheles fluviatilis s.l.*, *An. culicifacies s.l.*, *An. dthali,* and *An. superpictus s.l.* play the main role in malaria transmission in Hormozgan province [89]. Indeed, the environmental suitability of other vectors must be thoroughly assessed to gain a complete understanding of transmission dynamics. Moreover, malaria transmission is shaped by factors beyond vector presence in Iran, especially in this province. Effective malaria control requires implementing targeted interventions in high-risk areas, including preventing imported cases and identifying asymptomatic carriers [90,91]. Additionally, more research is needed to assess the shifting patterns of malaria transmission and adapt control strategies accordingly. This knowledge will enable the development of evidence-based vector control strategies. National and local malaria programs must proactively identify areas at risk of transmission to ensure effective intervention and sustainable disease control. In other words, effective malaria control strategies must go beyond vector surveillance and habitat assessment, incorporating socio-demographic factors and human movement patterns to develop more targeted interventions.

The analysis of variables underscores the importance of altitude and precipitation patterns in determining habitat suitability for *An. stephensi*. Moreover, response curve analysis further elucidates the complex relationships between environmental factors and species distribution. Altitude was identified as the most influential variable, suggesting that *An. stephensi* thrives in lower-altitude regions, which may explain its projected decline in coastal areas. Altitude plays a pivotal role, as species exhibit different responses at various altitudes. Following that, precipitation-related factors, particularly rainfall during the driest months, play a critical role, underscoring the sensitivity of *An. stephensi* to hydrological fluctuations. In this region, dry-season rainfall often occurs as flash floods, which subsequently create suitable breeding habitats for this vector. This is likely due to the formation of residual water pools after flooding, providing optimal conditions for oviposition and larval development. This discovery suggests that the optimal temperature and precipitation played a critical role in determining the spatial distribution of *An*. *stephensi*. *Anopheles* and *Plasmodium* are both influenced by temperature sensitivity. Given that mosquitoes are ectothermic organisms, temperature plays a crucial role in the developmental and mortality rates of each life stage [78,92]. Moreover, the gonotrophic cycle in adult female mosquitoes, which involves the blood meal-egg laying process, is also influenced by temperature fluctuations [93]. A notable correlation was observed between the monthly density of *An*. *stephensi* larvae and adults, and the levels of precipitation and mean temperature [94].

Recent studies have similarly identified key environmental factors influencing the distribution of malaria vectors, particularly *An. stephensi* [80,87]. These studies highlight that altitude, precipitation, and temperature are crucial variables impacting *An. stephensi* distribution [87]. Specifically, precipitation seasonality and annual mean temperature were noted as significant contributors to habitat suitability in Iran [80]. Similar modeling approaches for other *Anopheles* species, such as *An. farauti* in Australia has identified temperature, atmospheric moisture, and elevation as critical factors limiting coastal distribution [95]. This alignment with existing literature underscores the importance of altitude and precipitation in understanding the ecological niche of *An. stephensi*.

Moreover, isothermality (bio3) plays a crucial role in predicting *An. stephensi* distribution in the current study, as indicated by the significant decrease in model gain when it is omitted. This suggests that bio3 provides unique information that other climatic variables fail to capture. Isothermality represents the balance between daily and annual temperature fluctuations, which can directly impact the survival and adaptability of species, particularly *An. stephensi*. Our findings indicate that isothermality (bio3) values ranged between 32 and 44%, with the highest species response observed at 36%. It shows that the *An. stephensi* favors habitats where daily temperature fluctuations are relatively small compared to annual variations, possibly reducing thermal stress and enhancing survival rates. Given that climate change may alter temperature stability across different regions, shifts in bio3 could impact the species’ physiology [78]. *Anopheles* and *Plasmodium* are both influenced by temperature sensitivity. Given that mosquitoes are ectothermic organisms, temperature plays a crucial role in the developmental and mortality rates of each life stage [92,96]. Moreover, the gonotrophic cycle in adult female mosquitoes, which involves the blood meal-egg laying process, is also influenced by temperature fluctuations [93]. A notable correlation was observed between the monthly density of *An*. *stephensi* larvae and adults, and the levels of temperature [94]. A decrease in isothermality might expose the vector to harsher temperature fluctuations, potentially limiting its range, while an increase could create new suitable habitats. These results highlight the need to incorporate temperature stability into predictive modeling to improve our understanding of vector ecology and enhance malaria control strategies. Further research should explore how bio3 interacts with other climatic and ecological factors to refine species distribution predictions.

The Area Under the Curve (AUC) is a widely recognized metric in species distribution modeling, valued for its ability to assess predictive performance objectively [80,87]. In addition to AUC, the TSS was calculated to provide a complementary measure of model accuracy. In this study, the MaxEnt model demonstrated moderate predictive capacity for distinguishing suitable from unsuitable habitats for *An. stephensi*. This level of performance aligns with previous research findings [71,97–99], reinforcing the reliability of MaxEnt in ecological modeling. Various factors, including sample size, spatial resolution, choice of environmental predictors, and the method used for selecting pseudo-absence data, can influence the accuracy of predictive models. Refining these methodological aspects can help mitigate potential biases and enhance the reliability of distribution modeling. By optimizing data selection and model parameters, researchers can improve predictive performance and generate more precise insights for ecological and epidemiological applications [100,101].

Despite the valuable insights gained from this study, several limitations must be acknowledged. First, in presence-only species distribution modeling, pseudo-absence data are often used when true absence records are unavailable, providing a simple way to contrast presence points and prevent model convergence issues. However, the use of pseudo-absence data can introduce errors in predicted distributions, particularly if the selection of points does not accurately represent actual habitat conditions. This potentially can lead to over- or underestimation of the species’ range and reduce model accuracy, especially when presence data are unevenly distributed across geographic regions [100]. In this study, due to the limited availability of true absence data, background points were used, which is acknowledged as a limitation of the modeling approach.

Second, while the SSP scenarios provide a comprehensive look at potential future climates, they do not account for all possible local environmental changes, such as land-use alterations, which may further affect malaria vector distribution. Additionally, the study focuses primarily on one vector species, *An. stephensi*, while other species of *Anopheles* mosquitoes can influence their distribution through interspecific interactions or competition in the region [79]. Moreover, the dispersal of vectors can occur both naturally and through human-mediated transport. Natural dispersal is influenced by the flight capacity of mosquitoes, which can be incorporated into modeling frameworks to estimate local spread. Human-assisted dispersal, such as movement along roads, trade routes, or with transported goods, can facilitate long-distance introductions into new areas [102]. Incorporating these aspects into distribution models can enhance the spatial accuracy and predictive power of *An. stephensi* habitat suitability projections. Additionally, incorporating more detailed local environmental variables, such as land-use change and human infrastructure, would improve the model’s accuracy [103]. However, the primary scope of the present study was to assess the role of climatic conditions in shaping the potential ecological niches of *An. stephensi* in southern Iran. Due to the lack of reliable, spatially explicit data on human movement and public health interventions for this region, these factors could not be included in the current modeling framework.

Given the invasive nature of *An. stephensi*, it is also important to consider its capacity for further adaptation. Gene flow across populations could introduce novel heritable traits that enhance its ability to colonize diverse habitats. Population-genetic studies on *An. stephensi* and other malaria vectors (e.g., *An. gambiae*) have shown that even geographical barriers such as mountain ranges may not prevent gene flow, with genetic drift and human-mediated transport contributing to differentiation and spread [104,105]. Such findings emphasize that genetic connectivity and adaptive potential should be accounted for in long-term management strategies. In practical terms, longitudinal monitoring of distribution shifts in response to climate and anthropogenic changes will be invaluable for informing adaptive control measures. Regions such as Bashagard, which appear climatically stable in our projections, should be monitored closely as potential refuges for *An. stephensi*. Public health authorities may prioritize these areas for proactive surveillance.

### Conclusion

This study provides valuable insights into the impact of climate change on the distribution of *An. stephensi* in Hormozgan Province, Iran. Our findings highlight the potential decline in suitable habitats for this malaria vector under future climate scenarios, particularly in coastal areas. These changes underscore the urgency of adapting vector control strategies to the evolving environmental conditions. While the MaxEnt model offers strong predictive performance, further research is necessary to refine our understanding of how climate and ecological factors interact to shape vector populations. The findings emphasize the importance of monitoring malaria vectors continuously and integrating climate change projections into public health interventions. By doing so, we can better prepare for the challenges posed by climate change and protect vulnerable populations from malaria transmission.

## Supporting information

S1 Table Summary of *Anopheles stephensi* related data sources in Hormozgan province. (DOCX)

S1 File Occurrence records of *Anopheles stephensi* in Hormozgan Province, Iran (1970–2022). (CSV)
