# Supplementary material for: Suitable ecological niches of invasive malaria vector under present and projected climatic conditions in South of Iran
Source: PLoS Negl Trop Dis. 2026 Mar 16;20(3):e0014054. doi: 10.1371/journal.pntd.0014054 (PMC12991239; doi:10.1371/journal.pntd.0014054)
Supplement: S1 Table — (DOCX) [file pntd.0014054.s001.docx]

### S1 Table**: Summary of** *Anopheles* ***stephensi* Related Data Sources in Hormozgan Province**

| Author | Year | Source Title |
| --- | --- | --- |
| Manouchehri, AV. et al. (1) | 1976 | Ecology of *Anopheles stephensi* Liston in southern Iran |
| Edalat H, Yaghoobi‑Ershadi MR. (2) | 1977 | Propoxur selection of adults of *Anopheles stephensi* from Minab, south of Iran. |
| Ladonni, H. (3) | 1993 | Permethrin selection on the adults of *Anopheles stephensi* from south of Iran |
| Vatandoost H. et al. (4) | 2006 | Bionomics of *Anopheles stephensi* Liston in the malarious area of Hormozgan province, southern Iran |
| Vatandoost H. et al. (5) | 2004 | Larval habitats of malaria vectors and their susceptibility to larvicides in Hormozgan Province, Iran |
| Oshaghi, MA. et al (6) | 2006 | *Anopheles stephensi* Biological Forms; Geographical Distribution and Malaria Transmission in Malarious Regions of Iran |
| Soleimani Ahmadi, M. et al. (7) | 2011 | Field application of long-lasting insecticide-treated nets in Bashagard County, Hormozgan Province |
| Azizi K. et al. (8) | 2012 | Fauna and some biological characteristics of *Anopheles* mosquitoes (Diptera: Culicidae) in malaria high-risk regions: Hormozgan Province, 2007-2008 |
| Shahi, M. et al. (9) | 2013 | Susceptibility of *Anopheles stephensi* to deltamethrin and *Bacillus thuringiensis* |
| Majnoonpour, M.R. et al. (10) | 2014 | Fauna and distribution of Anopheline mosquitoes in Sirik county, Hormozgan province |
| Barati, M. et al. (11) | 2015 | Mapping and distribution of *Anopheles* species in Iran |
| Hatami-Gorbandi, J. et al. (12) | 2015 | Distribution of Anopheline Mosquitoes in Bandar Lengeh County, Hormozgan Province |
| Edalat, H. et al. (13) | 2015 | Vectorial Capacity and Age Determination of *Anopheles stephensi* Liston (Diptera: Culicidae) During the Malaria Transmission in Southern Iran |
| Abbassi, M. et al. (14) | 2019 | Resistance status of the main malaria vector, *Anopheles stephensi* Liston (Diptera: Culicidae) to insecticides in a malaria endemic area, Southern Iran |
| Vatandoost, H. et al. (15) | 2018 | Bioecology of Dominant Malaria Vector, *Anopheles superpictus* s.l. (Diptera: Culicidae) in Iran |
| Abbasi, M. et al. (16) | 2023 | Development of a degree-day model to predict the growth of Anopheles stephensi (Diptera: Culicidae): implication for vector control management. Environmental Entomology. |
| Sanei-Dehkordi, A. et al. (17) | 2019 | Species composition, seasonal abundance, and distribution of potential anopheline vectors in a malaria endemic area of Iran: field assessment for malaria elimination. |
| Abbassi, M. et al. (18) | 2020 | The impact of climatic variables on the population dynamics of the main malaria vector, Anopheles stephensi Liston (Diptera: Culicidae), in southern Iran |
| Salahi-Moghaddam, A. et al. (19) | 2022 | Spatio-temporal prediction of malaria transmission risk in Minab District, Hormozgan Province, Iran |
| Sanei-Dehkordi, A. et al. (20) | 2024 | Preliminary monitoring of knockdown resistance (kdr) mutation in *Anopheles stephensi* from Southeastern Iran |
| Nikpoor, F. et al (21) | 2024 | Long-lasting residual efficacy of Actellic®300CS and Icon®10CS on different surfaces against *Anopheles stephensi*, an invasive malaria vector |

### Reference:

1, Manouchehri AV, Javadian E, Eshighy N, Motabar M. Ecology of *Anopheles stephensi* Liston in southern Iran. Trop Geogr Med. 1976;28(3):228-32.

2. Edalat H, Yaghoobi‑Ershadi MR. Propoxur selection of adults of *Anopheles stephensi* from Minab, south of Iran. Iranian Journal of Public Health. 1997;26(3–4).

3, Ladonni H. Permethrin selection on adults of *Anopheles stephensi* from south of Iran. Iranian Journal of Public Health. 1993;22(1–4):11–22.

4. Vatandoost H, Oshaghi MA, Abaie MR, Shahi M, Yaaghoobi F, Baghaii M, et al. Bionomics of *Anopheles stephensi* Liston in the malarious area of Hormozgan province, southern Iran, 2002. Acta Trop. 2006;97(2):196-203.

5. Vatandoost H, Shahi H, Abai MR, Hanafi-Bojd AA, Oshaghi MA, Zamani G. Larval habitats of main malaria vectors in Hormozgan province and their susceptibility to different larvicides. Southeast Asian J Trop Med Public Health. 2004;35 Suppl 2:22-5.

6. Oshaghi M, F Y, Vatandoost H, Abai MR, Akbarzadeh K. *Anopheles stephensi* Biological Forms; Geographical Distribution and Malaria Transmission in Malarious Regions of Iran. Pakistan Journal of Biological Sciences. 2006;9. doi: 10.3923/pjbs.2006.294.298.

7. Soleimani-Ahmadi M, Vatandoost H, Shaeghi M, Raeisi A, Abedi F, Eshraghian MR, et al. Field evaluation of permethrin long-lasting insecticide treated nets (Olyset®) for malaria control in an endemic area, southeast of Iran. Acta Tropica. 2012;123(3):146-53.

8. Azizi K, Soltani A, mehranzadeh m, Poudat A. Fauna and some biologic characteristics of *Anopheles* mosquitoes (Diptera: Culicidae) in malaria high risk regions: Hormozgan Province, 2007-2008. Bimonthly Journal of Hormozgan University of Medical Sciences. 2011;16:273-82.

99. SHahi M, Hanafi-bajd AA, Vatandost H, Soleimani-Ahmadi M. Susceptibility Status of *Anopheles Stephensi* Liston the Main Malaria Vector, to Deltamethrin and Bacillus Thuringiensis in the Endemic Malarious Area of Hormozgan Province, Southern Iran. Journal of Kerman University of Medical Sciences. 2012;19(1):87-95.

10. Majnoonpour MR, Soleimani-Ahmadi M, Poorahmad-Garbandi F. Fauna and distribution of Anopheleline mosquitoes in Sirik county, Hormozgan province, 2014. hums-jpm. 2015;2(1):29.

11. Barati M, Khoshdel A, Sedaghat Mm, Salahi-Moghaddam A. An overview and mapping of *Anopheles* in Iran. ajaums-jps. 2015;10(1):9.

12. Hatami-Gorbandi J, Poorahmad-Garbandi F, Soleimani-Ahmadi M, Sanei-Dehkordi A. Distribution of Anopheleline mosquitoes in Bandar Lengeh County, Hormozgan provice. Journal of Preventive Medicine. 2015;2(4):21-8.

13. Edalat H, Moosa‑Kazemi SH, Abolghasemi E, Khairandish S. Vectorial capacity and age determination of *Anopheles stephensi* Liston (Diptera: Culicidae) during malaria transmission in southern Iran. Journal of Entomology and Zoology Studies. 2015;3(1):256–263.

14. Abbasi M, Hanafi-Bojd AA, Yaghoobi-Ershadi MR, Vatandoost H, Oshaghi MA, Hazratian T, et al. Resistance status of main malaria vector, *Anopheles stephensi* Liston (Diptera: Culicidae) to insecticides in a malaria Endemic Area, Southern Iran. Asian Pacific Journal of Tropical Medicine. 2019;12(1).

15. Vatandoost H, Hanafi-Bojd AA, Raeisi A, Abai MR, Nikpour F. Bioecology of Dominant Malaria Vector, *Anopheles superpictus s.l.* (Diptera: Culicidae) in Iran. J Arthropod Borne Dis. 2018;12(3):196-218.

16. Abbasi M, Oshaghi MA, Mehdi Sedaghat M, Hazratian T, Rahimi Foroushani A, Jafari-Koshki T, et al. Development of a degree-day model to predict the growth of *Anopheles stephensi* (Diptera: Culicidae): implication for vector control management. Environmental Entomology. 2023;52(6):1126-38. doi: 10.1093/ee/nvad092.

17. Sanei-Dehkordi A, Soleimani-Ahmadi M, Jaberhashemi SA, Zare M. Species composition, seasonal abundance and distribution of potential anopheline vectors in a malaria endemic area of Iran: field assessment for malaria elimination. Malar J. 2019;18(1):157.

18. Abbasi M, Foroushani AR, Jafari-Koshki T, Pakdad K, Vatandoost H, Hanafi-Bojd AA. The impact of climatic variables on the population dynamics of the main malaria vector, *Anopheles stephensi* Liston (Diptera: Culicidae), in southern Iran. Asian Pacific Journal of Tropical Medicine. 2020;13(10):448-55. doi: 10.4103/1995-7645.291038. PubMed PMID: 01542552-202013100-00004.

### 19. Salahi-Moghaddam A, Turki H, Yeryan M, Fuentes MV. Spatio-temporal Prediction of the Malaria Transmission Risk in Minab District (Hormozgan Province, Southern Iran). Acta Parasitologica. 2022;67(4):1500-13. doi: 10.1007/s11686-022-00598-2.

20. Sanei-Dehkordi A, Paksa A, Gorouhi MA, Soleimani-Ahmadi M, Jaberhashemi SA, Salim Abadi Y. Preliminary monitoring of knockdown resistance (kdr) mutation in *Anopheles stephensi*: insights from a malarious area in Southeastern Iran. Malar J. 2024;23(1):211.

### 21. Nikpour F, Vatandoost H, Hanafi-Bojd AA, Raeisi A, Mirolyaie A, Mojahedi AR, et al. Long-lasting residual efficacy of Actellic300CS and Icon10CS on different surfaces against *Anopheles stephensi*, an invasive malaria vector. Trop Med Int Health. 2024;29(9):781-91. Epub 20240731. doi: 10.1111/tmi.14028. PubMed PMID: 39081142.
